# Readiness for Integrated Care of Older People: A Cross-Sectional Study in Mexico

**DOI:** 10.7759/cureus.49646

**Published:** 2023-11-29

**Authors:** Juan Pablo Gutierrez, Arturo Avila-Avila, Eduardo Sosa-Tinoco, Luis Miguel Gutierrez-Robledo, Sergio Flores-Hernández, Samuel E Gutierrez-Barreto

**Affiliations:** 1 Epidemiology and Public Health, Center for Policy, Population and Health Research, National Autonomous University of Mexico, Mexico City, MEX; 2 Geriatrics, National Institute of Geriatrics, Mexico City, MEX; 3 Geriatrics, Health Systems Research Center, National Institute of Public Health, Mexico City, MEX; 4 Medical and Health Sciences, National Autonomous University of Mexico, Mexico City, MEX

**Keywords:** older aged people, quality improvement (qi), health program evaluation, musiq, icope

## Abstract

Aim: To evaluate the readiness of the Mexican healthcare system to implement the integrated care for older people (ICOPE) approach into an existing healthcare model.

Methods: We conducted a cross-sectional study with data from 2473 healthcare workers analyzed using the model for understanding success in quality (MUSIQ) framework to gather data from healthcare professionals. Their perceptions regarding the readiness for ICOPE were assessed across five dimensions: team, microsystem, infrastructure, organization, and external environment.

Results: Only 717 (29%) of the participants believed ICOPE could be successfully implemented in Mexico without any modifications. A total of 1261 (51%) participants rated the readiness of ICOPE with some barriers. The main barriers were reallocating resources and the external environment.

Observation: Mexico's healthcare system faces barriers to innovation that could hinder the successful integration of the ICOPE approach. A systematic identification of these barriers provides an opportunity to suggest adaptations and refinements to increase the probability of success. Using the contextual factors identified as facilitators and the proposal of interventions such as the ICOPE app could improve the chances of success.

Conclusion: The participants of this study evaluated ICOPE as ready to implement, with some contextual barriers. The readiness evaluation supports the stakeholders' and policymakers' decisions in implementing and monitoring the program in a natural setting. Evaluating the readiness of the intervention increases the possibility of aligning the innovation with contextual factors, increasing the chances of its successful adoption and implementation.

## Introduction

Population aging worldwide is challenging health systems' capacity to provide adequate care to the elderly, as most lack healthcare models that include the needs of this age group [1]. For health systems to fulfill their mission of maintaining the health of their populations, ensuring the quality of healthcare is critical [2]. Currently, healthcare services are ill-prepared to address the complexity of elders' health needs: increased morbidity, high prevalence of cognitive impairment, depressive symptoms, and dependence, threatening their quality of care [1,3]. There is evidence that healthcare systems in lower-middle-income countries provide low-quality care, leading to increased mortality and morbidity [4].

While low-quality healthcare affects everyone, older people (OP) are particularly affected by poor quality because of their increased need for healthcare and the lack of a model that addresses their needs [5]. Older people (65 years and older) represent about 10% of the total population worldwide and 8% in Mexico; this share is expected to double by 2030 [6]. Health workers must address the healthcare needs of the elderly and thus require a healthcare model that includes the specific needs of this population.

A promising approach to this end is a framework to implement integrated care and provide healthcare workers (HCWs) with support systems for evidence-based clinical decision-making [7]. Within the framework of the Decade of Healthy Aging 2020-2030, the WHO has proposed the integrated care of older people (ICOPE) approach as a group of guidelines to implement comprehensive and coordinated primary healthcare approaches [8]. The ICOPE program provides a framework to align health systems to the diverse needs of the aging population [9]. This framework promotes person-centered care by measuring intrinsic capacity, a new concept defined by WHO in the Plan of Action 2016-2020 as "the composite of all the physical, functional, and mental capacities of an individual" [9]. This concept focuses on disease prevention, which is related to an individual's ability to overcome stressful or acute events [10,11]. The WHO reported that the ICOPE is feasible to implement in different countries and contexts with proper preparation and adaptation [12]; a comprehensive understanding of the context is needed to inform such preparation and adaptation. Therefore, we aim to evaluate the readiness of the Mexican healthcare system to implement the ICOPE approach into its existing healthcare model.

## Materials and methods

Study design and participants

We followed the 'strengthening the reporting of observational studies in epidemiology' (STROBE) guidelines for reporting cross-sectional observational studies [13]. This study was reviewed by the Ethics Board of the School of Medicine, National Autonomous University of Mexico (approval no. PMDCMOS/CE4/02/2021). We performed a cross-sectional study, with data collected from January to December 2021 from participants in a series of implementations of a massive online open course (MOOC) about ICOPE designed and implemented by the National Geriatrics Institute of Mexico. The MOOC was open to people interested in OP care. This course was disseminated online and made available free of charge to all interested HCWs.

The Mexican health system consists of three main components operating in parallel: 1) employment-based social insurance schemes; 2) public assistance services for the uninsured supported by financial protection schemes; and 3) a private sector composed of service providers [14]. After completing the course, we presented participants with an online written explanation of the study and asked for their informed consent to participate. For those who provided consent, the platform presented the study questionnaire, which included sociodemographic data. Participants answered the online questionnaire without time control, supervision, or consequences in the responses to avoid biased responses.

Study variables

The questionnaire used the quantitative tool for the Model for Understanding Success in Quality (MUSIQ) framework that assesses the contextual factors before implementation [15-17]. The MUSIQ framework has 24 contextual factors divided into six context dimensions defined in Table 1 [16,18] that explore the roles and inter-relationships between contextual factors within the quality improvement and implementation programs. With the MUSIQ framework, HCWs assess their context to implement ICOPE by identifying gaps and adjusting ICOPE to the context. We adapted the instrument to use the MUSIQ tool for this study because there is no formal quality improvement (QI) team in Mexico's community healthcare sub-system. In consensus, the authors of this study excluded the questions that explore the QI dimension; the contextual factors were team subject matter expert, QI team diversity, and QI team leadership. The rest of the questions were adapted to reflect the implementation of ICOPE.

**Table 1:** Definitions of contextual factors according to MUSIQ 2.0 Adapted from Reed et al. [14] MUSIQ: Model for understanding success in quality, QI: Quality improvement

Dimension	Contextual factor	Definition
1 . Team	Team permanence	Team members have worked together as a team before
	Team skills	Team's ability to use improvement methods to make changes
	Norms	The team establishes strong norms of behavior related to how work is conducted and how goals are to be achieved.
2. Microsystem	Microsystem team culture	Values, beliefs, and norms present in the microsystem that emphasize teamwork, communication, freedom to make decisions, and commitment to improve
	Microsystem team capability	Microsystem staff's ability to use QI methods for change
	Microsystem motivation	The extent to which microsystem staff members have a desire and willingness to improve performance in this area of focus
	Microsystem leaders	Top managers with responsibility for the operation and administration of the microsystem affected by this project. Microsystem leaders may include department or division chairs, department managers, ward/unit medical or nursing directors, business unit managers, and a senior physician in a large physician group.
3 . Infrastructure	Data infrastructure	The extent to which a system exists to collect, manage, and facilitate the use of data needed to support performance improvement
	Resource availability	The degree to which financial support is provided for QI, including allocation of resources and staff time.
4 . Organization	Task strategic importance to the organization	Work perceived as part of the organization's strategic goals
	Workforce focus	The degree to which the organization develops the workforce through training and engages them in QI through reward systems and expectation-setting
	Maturity	The sophistication of the organization's QI program
	Organizational culture	Values, beliefs, and norms of an organization that shape the behaviors of staff in pursuing QI
	The senior leader project sponsor	A senior leader committed to championing and supporting the project
	Organizational leadership	Senior management's governance—guidance, support, oversight, and direction-setting—of improvement efforts
5. External environment	Triggering event	Presence of a specific event (positive or negative) that stimulates a new emphasis on improving quality in the focus of a given project
	External project sponsorship	Substantial and meaningful contributions of personnel, expertise, money, equipment, facilities, or other essential resources from outside entities (external to the organization) with formal relationships with this QI project
	External motivators	Environmental pressures and incentives that stimulate the organization to improve its performance and quality around the focus of the project

Then, we followed the RAND criteria to translate the questionnaire into Spanish. In Table 1, we describe, according to MUSIQ, the dimensions and contextual factors used in the instrument. We used the original Likert-type scale responses from 7 to 0 (totally agree to totally disagree, respectively, and zero for do not know), which are interpreted as follows: 100% is the highest possible MUSIQ score; 99% to 75% indicates the project has a reasonable chance of success; 74% to 50% implies the project could be successful but has potential contextual barriers; 49% to 25% suggests the project has severe contextual issues and is not set up for success; 25% to 14% means the project should not continue as is and resource deployment to other improvement activities must be considered; 14% to 2% is the lowest [16,17] possible MUSIQ score when all questions are answered; and 1% the lowest possible MUSIQ score. Following this, the HCWs answered the adapted MUSIQ instrument (see Appendix A), which included 23 items with 18 contextual factors.

Participants' sociodemographic characteristics included age, sex, place of work, profession, and state of residence. Participants were grouped by their institution of work sector (private or public) and profession (physician, other health professionals, or non-health professionals). They were also grouped per the economic region of their residence state (North: Baja California, Baja California Sur, Chihuahua, Coahuila, Nuevo León, Sinaloa, Sonora y Tamaulipas; Central-North: Aguascalientes, Durango, San Luis Potosí y Zacatecas; Center: Ciudad de México, Estado de México, Guanajuato, Hidalgo, Morelos, Puebla, Querétaro y Tlaxcala; Western: Colima, Jalisco, Michoacán y Nayarit; South: Chiapas, Guerrero y Oaxaca; and, Southeast: Campeche, Quintana Roo, Tabasco, Veracruz y Yucatán) [19].

Statistical analyses

We analyzed the data with JASP 0.17.1. The report includes means and standard deviations for continuous variables and absolute and relative frequencies for categorical variables in the descriptive analyses. The differences between the sociodemographic variables and the means for the overall results were assessed with the chi-square test. Since no samples showed the prevalence of readiness using MUSIQ, we conservatively assumed 50%. We used the OpenEpi online application to calculate the required sample proportions with the following parameters: alpha = 0.01 and power = 0.80 [20]. The estimated sample size was 1483.

## Results

All 2473 registered HCWs who completed the ICOPE training agreed to answer all the questions, and 1892 (76.5%) participants agreed to share their sociodemographic characteristics (Table 2). Participants' average age was 37 ±10, and 78.5% (1485) were women. Participants were from public (79.4%, 1502) and private (20.6%, 390) services and were from all 32 states in Mexico. In terms of their professional backgrounds, 32.6% (617) of participants were physicians, 43.1% (815) were from other health professions (nurses, nutritionists, gerontologists, health promoters, dentists, and phycologists), and 24.3% (460) were from non-direct health professions (administrators, policymakers, stakeholders, and social workers). Most participants (93.4%, 1767) were direct care practitioners working directly with the OP (nurses, nutritionists, social workers, gerontologists, health promoters, dentists, and phycologists), while the remaining had management functions.

**Table 2:** Sociodemographic information of the participants (n=1892)

Variables	All n (%)	Physicians n (%)	Other health professions n (%)	Non-health profession n (%)
Sex				
Female	1485 (78.5)	415 (21.9)	688 (36.4)	382 (20.2)
Male	407 (21.5)	201 (10.6)	129 (6.8)	77 (4.1)
Health services				
Public	1502 (79.4%)	533 (28.2)	637 (33.7)	332 (17.5)
Private	390 (20.6%)	83 (4.4)	180 (9.5)	127 (6.7)
Region				
North	265 (14%)	98 (5.2)	89 (4.7)	78 (4.1)
Central-North	162 (8.6%)	66 (3.5)	71 (3.8)	25 (1.3)
Central	886 (46.8%)	283 (15)	391 (20.7)	212 (11.2)
Western	198 (10.5%)	54 (2.9)	92 (4.9)	52 (2.7)
Southern	171 (9%)	45 (2.4)	64 (3.4)	62 (3.3)
Southeast	210 (11.1%)	70 (3.7)	110 (5.8)	30 (1.6)
Employee				
Direct care practitioners	1767 (93.4)	602 (31.8)	771 (40.8)	394 (20.8)
Manager	125 (6.6)	14 (0.7)	46 (2.4)	65 (3.4)
Total		616 (32.6%)	817 (43.1%)	459 (24.3%)

**Figure 1:**
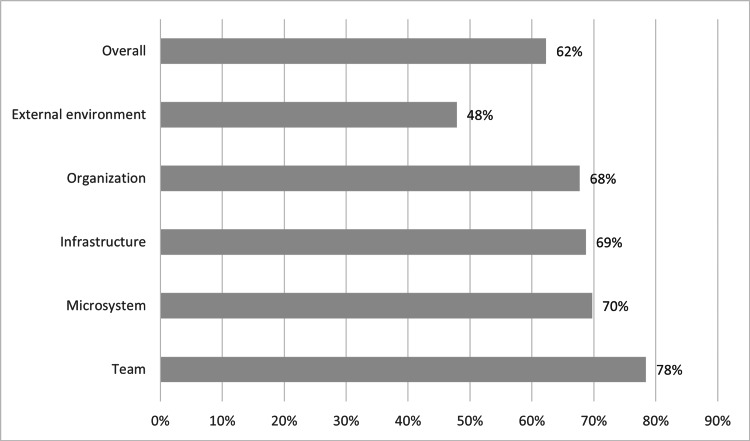
Dimensions of the global results of the MUSIQ score (n=2473) MUSIQ: Model for understanding success in quality

In Figure 1, we report the mean scores of overall MUSIQ and the respective dimensions. It shows that the lower score for the external environment dimension (48%) implies not being set up for success and having severe contextual issues. The dimensions of the organization (68%), infrastructure (69%), and microsystem (70%) resulted in the ICOPE being successful with possible contextual barriers. Finally, the team dimension (78%) had a score of a reasonable chance of success. The overall score was 62%, showing that ICOPE could be successful.

In Table 3, we report the scores for the contextual factors by dimension and contextual factor. The dimensions with the highest standard deviation were the external environment (2.1), and the lowest was the team (1.75). The contextual factors averaged with the interpretation of the ICOPE project have a reasonable chance of success: norms (6.02), microsystem team culture (5.67), strategic task importance to the organization (5.32), and maturity (5.63). Then the interpretation, as ICOPE has some contextual barriers, was team permanence (4.45), team skills (4.96), microsystem team capability (4.28), microsystem motivation (4.80), microsystem leaders (4.83), data infrastructure (5.16), resource availability (4.46), workforce focus (4.63), senior leader project sponsors (5.24), organizational leadership (5.05), and external motivators (4.11). Finally, the factors interpreted as ICOPE having serious contextual barriers were organizational culture (2.91), triggering events (3.35), and external project sponsorship (2.59). Table 4 compares the total score by sex, public vs. private practice, profile (physicians, health-related, and non-health professions), and region. As observed, no relevant differences were identified between sub-groups.

**Table 3:** Results for the contextual factors (n=2473) *Average was calculated from 0 to 7

Dimension Mean (SD)	Contextual Factor		Factor Average* (SD)
Team 5.49 (1.75)	Team permanence		4.45 (2.43)
	Team Skills		4.96 (2.13)
	Norms		6.02 (1.63)
Microsystem 4.88 (1.8)	Microsystem team culture		5.67 (1.88)
	Microsystem team capability		4.28 (2.29)
	Microsystem motivation		4.80 (2.37)
	Microsystem leaders		4.83 (2.36)
Infrastructure 4.81 (1.9)	Data infrastructure		5.16 (2.13)
	Resource availability		4.46 (2.43)
Organization 4.74 (1.49)	Task strategic importance to the organization		5.32 (2.27)
	Workforce focus		4.63 (2.11)
	Maturity		5.63 (1.89)
	Organizational culture		2.91 (2.36)
	Senior leader project sponsor		5.24 (2.43)
	Organizational leadership		5.05 (2.37)
External environment 3.35 (2.1)	Triggering event		3.35 (2.37)
	External project sponsorship		2.59 (2.27)
	External motivators		4.11 (2.37)
Total mean 4.36 (1.29)			

**Table 4:** Mean of the MUSIQ score by sex, type of practice, and region (n=1892) MUSIQ: Model for understanding success in quality

Variables	Mean (n)
Sex (p >0.300)	
Female	4.3 (1485)
Male	4.4 (407)
Health services (p >0.027)	
Public	4.4 (1505)
Private	4.3 (390)
Region	p 0.233
North	4.4 (265)
Central-North	4.4 (162)
Central	4.3 (886)
Western	4.3 (198)
South	4.6 (171)
Southeast	4.3 (210)
Profile (p >0.071)	
Physicians	4.3 (616)
Other health professions	4.4 (817)
No health-profession	4.4 (459)
Employee (p >0.076)	
Direct care practitioners	4.3 (1767)
Manager	4.7 (125)

## Discussion

Our study's findings, as indicated by the overall MUSIQ score, suggest that while the ICOPE approach could be implemented successfully in Mexico, there are significant barriers to consider. The overall score of MUSIQ was that 51% of the participants scored that ICOPE could be implemented successfully in Mexico, with some barriers, such as reallocating resources. The contextual factors identified could be addressed in a community setting to improve the success probability. The ICOPE is a potentially feasible intervention that requires adaptation and refinement to address barriers.

Understanding the context for innovation implementation is especially critical when their success depends on the operation setting when the implementation relies heavily on the existing staff and operational procedure arrangements. Frameworks that allow disentangling relevant factors of the innovation context, such as MUSIQ, allow gathering preliminary information on the implementation process from those in charge of operating or evaluating it [21]. The advantage of using MUSIQ to evaluate context is that it is a pre-tested, evidence-based framework [15]. The questionnaire used to explore the context through MUSIQ was adapted to reflect the lack of a QI team in the community setting, like other adaptations of the MUSIQ [16,22]. Programs such as QI interventions may need to assess their context and work to address shortcomings before the intervention is fully implemented. The evidence suggests a critical link exists between evidence for barriers and facilitators to integrated care approaches for OP across different contexts [23].

The relatively high scores in the team dimension (78%) and microsystem (70%) suggest that the HCWs in Mexico have a reasonable understanding and capability to work as a team, which is crucial for implementing ICOPE. The strong team norms, microsystem team culture, and leadership indicate a conducive environment for collaborative efforts, a critical component of the ICOPE program. One contextual factor that is considered a barrier is the lack of resources; increasing the availability of electronic devices could be a way to ease this barrier in our and similar contexts [24]. It is crucial to address resource availability before implementation. Directing resources for ICOPE requires budget reallocation, which implies high-level decision-making. The ICOPE mobile application mitigates the gap between specialists and community HCWs [24]. Using a mobile application such as the ICOPE app might be helpful in robusting the data infrastructure. The ICOPE app applies the "action-research philosophy" and provides a systematic assessment to bridge the gap between research and clinical practice and provide better care for the older community [25]. It is crucial to build an investment case using demographic data to highlight that providing adequate care for the OP with a preventive approach will help contain costs and improve the chances of success. However, in our results, one contextual factor considered a possible barrier was the data infrastructure, which has a substantial cost to the health systems. When funders are required to help with the cost of any implementation, they should provide guidance and expertise in measurement, data collection, and analytic strategies [25]. Starting any program requires an evaluation process to increase its likelihood of success. Also, HCWs must be taught how to use the ICOPE app or similar tools to ensure implementation [26]. Possibly, this was specific to the study conducted in a particular training program and not in a specific setting. Context evaluation is helpful in an organization to fulfill the facilitator. An organization adopting a context-appropriate implementation strategy can change the outcome by triggering enablers [22].

The organizational culture poses a significant barrier, likely due to resistance to changing the status quo. This challenge underscores the need for a cultural shift within healthcare organizations to embrace the ICOPE program's preventive and comprehensive care model. Utilization of the existing robust team dynamics and microsystem culture to foster collaborative ICOPE implementation efforts is essential. Another dimension that scored low was the external environment. Participants were uncertain of the level of support that ICOPE could get from the high-level managers. One path to implementing ICOPE on a large scale in Mexico could be a concept-proof-type implementation, i.e., generating the evidence that it is feasible to operate the ICOPE approach within the existing model. This proof-type implementation will generate the needed support. The organizational culture had a low score; the challenges of the status quo of the HCWs could explain this. At the organizational level, it is relevant to demonstrate the feasibility of the model and its benefits. The organizational culture marked as a barrier may be related to the ICOPE program's focus on improving intrinsic capacity. Some studies have demonstrated that health improvement programs do little to improve specific health outcomes [24]. This factor could be a facilitator if the perspective focuses on improving health services, as incorporated in Mexico's OP health vaccination card.

Understanding how frameworks related to context can be utilized in the real world would benefit public health evaluators and implementers in their work [21]. Program evaluation could help identify the effectiveness of interventions targeted at a broader range of clinical, professional, organizational, and system levels of care [23]. Contextual elements, which changed over time, influenced the success of the implementation at the micro level. However, at the meso level, the organization has not matured for systemic implementation of the method [22].

We suggest implementing ICOPE in contexts similar to ours, which requires an agent to guide the intervention, an electronic application, and matching the reference system with the one proposed by the WHO. With the analyses of the context and the correlations of dimensions, the previous interventions could improve the chances of implementing ICOPE in a lower-middle-income country. One of the limitations of this study was the validation of the MUSIQ adaptation. However, some questions were eliminated, and the rest were used, like the original questionnaire [16]. The sample was only for the HCWs who took the ICOPE course but included several HCWs with diverse backgrounds and in all states of Mexico; these variations could be a strength in the context of understanding this sample. The results of this study can be used in similar contexts, i.e., resource-constraint settings and without a QI team. Further study could be a program evaluation to address all the facilitators and barriers in the context and improve the chances of success of this QI intervention.

## Conclusions

While there are challenges to implementing the ICOPE approach in Mexico, our study reveals that the potential for success exists, provided strategic efforts are made to address the key barriers and leverage existing strengths within the healthcare system. The readiness evaluation supports the stakeholders' and policymakers' decisions in implementing and monitoring the program in a natural setting. Also, it helps understand the relationships between the factors that serve as facilitators and barriers. Evaluating the readiness of the intervention increases the possibility of aligning the innovation with contextual factors, increasing the chances of its successful adoption and implementation. Using an evidence-based framework to assess the contextual factors before implementing ICOPE could drive efforts to improve its chances of success. The assessment of the context setting assists the discussion on how to manage the factors and the setting to improve the health system and services. In this study, the team dimension indicates a high probability of success for the ICOPE approach. In contrast, the scores of the organization and external environment dimensions represent a threat to the feasibility of the ICOPE implementation. These barriers must be addressed in the design of the intervention to scale up the performance of the ICOPE approach.

## Appendices

Appendix A

MUSIQ Instrument Adaptation

The following was adapted from the study by Reed et al. [14].

1. Will ICOPE's overall goals guide the task force's activities to improve the quality of care? (QI team)

2. Will my team members behave as they are expected to act in the ICOPE application? (QI team)

3. Are all team members committed to the same ICOPE goals? (QI team)

4. Does the team where I work effectively use quality improvement methods (Plan-Do-Study-Act cycles, execution charts, control charts) to make changes in attention? (QI team)

5. Were the team members I work with familiar with each other before I started this program? (QI team)

6. Do the unit leaders personally facilitate the implementation of ICOPE? (Microsystem)

7. Does my unit value teamwork, communication, and commitment to quality improvement? (Microsystem)

8. Are staff not aware of ICOPE effectively identifying the functions of screening, evaluating, and developing personalized care plans? (Microsystem)

9. Do non-operational staff have a strong desire to improve performance in care with ICOPE? (Microsystem)

10. Do existing information systems allow us to extract data to know if ICOPE usage works efficiently? (Infrastructure)

11. Do the operational staff have adequate resources and time to meet ICOPE objectives? (Infrastructure)

12. Would the managers of my unit be directly involved in the quality improvement activities implementing ICOPE? (Organization)

13. Does at least one head of unit support staff specifically for ICOPE implementation? (Organization)

14. Does the unit you work in NOT value quality improvement with ICOPE? (Organization)

15. Is quality improvement part of the culture in your unit?

16. Do my unit staff receive education and training on identifying and acting on opportunities to improve care quality, such as ICOPE? (Organization)

17. Do my unit staff receive education and training in statistical and other quantitative methods that support improving the quality of care? (Organization)

18. Do my unit staff receive the education and training necessary to improve their job skills and performance? (Organization)

19. Are staff rewarded and recognized for improving the quality of care? (Organization)

20. Is ICOPE directly aligned with the organization's key strategic objectives? (Organization)

21. Did the pressures or incentives external to my organization motivate us to undertake this project on ICOPE? (Environment)

22. Do groups outside my organization provide the necessary staff, money, resources, or training to support ICOPE? (Environment)

23. Did a specific event prompt the launch of ICOPE? (Triggering event)
